# Local-Scale Diversity and Between-Year “Frozen Evolution” of Avian Influenza A Viruses in Nature

**DOI:** 10.1371/journal.pone.0103053

**Published:** 2014-07-30

**Authors:** Alexander Nagy, Lenka Černíková, Helena Jiřincová, Martina Havlíčková, Jitka Horníčková

**Affiliations:** 1 State Veterinary Institute Prague, National Reference Laboratory for Avian Influenza and Newcastle Disease, Laboratory of Molecular Methods, Prague, Czech Republic; 2 National Institute of Public Health, Centre for Epidemiology and Microbiology, National Reference Laboratory for Influenza, Prague, Czech Republic; Friedrich-Loeffler-Institut, Germany

## Abstract

Influenza A virus (IAV) in wild bird reservoir hosts is characterized by the perpetuation in a plethora of subtype and genotype constellations. Multiyear monitoring studies carried out during the last two decades worldwide have provided a large body of knowledge regarding the ecology of IAV in wild birds. Nevertheless, other issues of avian IAV evolution have not been fully elucidated, such as the complexity and dynamics of genetic interactions between the co-circulating IAV genomes taking place at a local-scale level or the phenomenon of frozen evolution. We investigated the IAV diversity in a mallard population residing in a single pond in the Czech Republic. Despite the relative small number of samples collected, remarkable heterogeneity was revealed with four different IAV subtype combinations, H6N2, H6N9, H11N2, and H11N9, and six genomic constellations in co-circulation. Moreover, the H6, H11, and N2 segments belonged to two distinguishable sub-lineages. A reconstruction of the pattern of genetic reassortment revealed direct parent-progeny relationships between the H6N2, H11N9 and H6N9 viruses. Interestingly the IAV, with the H6N9 subtype, was re-detected a year later in a genetically unchanged form in the close proximity of the original sampling locality. The almost absolute nucleotide sequence identity of all the respective genomic segments between the two H6N9 viruses indicates frozen evolution as a result of prolonged conservation in the environment. The persistence of the H6N9 IAV in various abiotic and biotic environmental components was also discussed.

## Introduction

Influenza A virus (IAV) is a member of the genus *Orthomyxoviridae* with a genome composed of eight distinct negative-sense RNA segments. The segmented genome and the lack of proofreading activity of the virus RNA polymerase provide a basis for extreme genetic diversity.

Monitoring studies carried out during the last two decades provided a large body of knowledge regarding the ecology of IAV in wild birds. Multiyear studies utilizing data from various bird populations sampled in different areas worldwide allowed us to identify the reservoir bird species and revealed the main ecological characteristics like the prevalence, subtype diversity, seasonality, environmental persistence, transmission routes, geographic distribution, intercontinental exchange, etc. [1]–[6]. Comprehensive evolutional analyses of the IAV genome have suggested extensive and ubiquitous reassortment [7], [8] and rapid evolutionary dynamics in the avian reservoir [9].

Nevertheless, there are still aspects of IAV evolution and ecology in wild birds which have remained to be elucidated. For example, contrary to the IAV prevalence and subtype variety reported in the monitoring studies, only a few projects were focused on revealing the subtype diversity, genomic complexity, and dynamics of genetic interactions between the viruses occurring at the local-scale level, i.e. in the wild bird population residing in a single locality or in a single pond [10]–[12].

Another sparsely reported and not fully understood issue is the phenomenon of “frozen evolution” of the influenza virus in nature. The hypothesis of frozen evolution or frozen replication (both terms are used in the literature) is used to explain occasional evidence of anachronistic influenza virus genomes or genomic segments [13]–[16]. Anachronistic sequences exhibit unusually high or absolute sequence identity at the nucleotide level despite relative distance in reported time of detection. Therefore, it appears that they are being “frozen in time”. This is the main difference to the hypothesis of evolutionary stasis [1], [17]. According to this hypothesis the IAV virus proteins are under strong purifying selection in avian reservoir as a result of adaptive optimum. Nevertheless, continuous circulation in the wild bird population results to continuous accumulation of nucleotide changes. However, from of these changes the synonymous mutations are selected predominantly. Hence, purifying selection results to circulation of phenotypically equivalent virus proteins [8] which appears to be at apparent stasis, despite the continuous accumulation of mutations at the nucleic acid level. Taking together, the two hypotheses relates to two different levels. Frozen evolution relates to the nucleotide (genotype) while the evolutionary stasis to the amino acid (phenotype) conservation respectively.

So, the frozen evolution is a hypothesis explaining significantly lover nucleotide mutation rate than expected [9]. The mechanism of frozen evolution therefore requires some kind of environmental persistence without the ability to replicate, accumulate nucleotide changes, and evolve. However, others consider anachronistic sequences as laboratory artifacts [18], [19]. Therefore, the existence and operation of IAV frozen evolution in nature is unclear.

The objective of our study was to help to elucidate these aspects of IAV evolution by investigating the genetic diversity and evolutionary relationships between the viruses detected one year apart from two neighboring localities. In 2009, unusually high IAV subtype diversity was observed in a sample pool from a mallard flock inhabiting a pond in the South Bohemian Region in the Czech Republic. A year after, the viruses collected in the vicinity of the first locality exhibited exceptionally high sequence similarity to that of the previously identified IAV strains. South Bohemia is known for its countless ponds and lakes which have been established since the 12^th^ century. The ponds are often interconnected with streams into cascades resulting to a dense network of pond and lake systems through the landscape the role of which in the ecology and perpetuation of the IAV has not been fully investigated yet.

We analyzed the subtype diversity of AIV in each of the study localities. Further, the eight genomic segments of the co-circulating virus strains were examined to determine the nucleic and amino acid sequence diversity, genotype constellations, and patterns of genetic reassortment. Finally, we focused on the estimation of the evolutionary relationships between the viruses from the two localities.

The phylogenetic analysis and genotyping revealed remarkable genomic variability, with the identification of direct parent-progeny relationships between the newly emerging IAV genotypes. In addition, mutual comparison of IAV from the two localities indicated between-year frozen evolution.

## Materials and Methods

### Virus detection and isolation

Cloacal and tracheal swabs (147C Virus Transport-Single Swab, Copan Innovation, Italy) were collected post mortem from hunting harvested mallards (Anas platyrhynchos) during the National avian IAV surveillance in the Czech Republic in 2009 and 2010. As the National surveillance program no specific permissions were required to access the sampling localities and for sampling activities. The sample collection was coordinated with the Regional Veterinary Administration of the Czech Republic and the given hunting organizations. Our study did not involve endangered or protected species. All the specimens were collected post mortem after the hunting harvest. The GPS coordinates of the sampling localities were provided in Figure S1 in File S1. The birds were not shot for the purpose of our study. No specific hunting permission was required to hunt the animals used in this study.

The swabs were re-suspended in PBS buffer and the suspensions were then divided into aliquots and used either for molecular detection or virus isolation. Total nucleic acid was extracted using the MagNA Pure Compact and MagNA Pure LC extractors (Roche), employing the Total Nucleic Acid Extraction Kit (Roche) with an input volume of 200 or 400 µl and an elution volume of 50 µl. The extracts were screened for IAV by the RT-qPCR method of Nagy et al. [20]. Subsequently, the IAV-positive specimens were tested for all nine NA subtypes (OneStep RT–PCR kit, Qiagen) [21] and for the most common HA subtypes: H5, H7, H9 (QuantiTect Probe RT–PCR kit, Qiagen) [22], [23], and H3, H4, H6, and H11 (OneStep RT–PCR kit, Qiagen; the primer sets are available on request). In addition, a universal HA typing approach was applied [24]. The results of conventional RT-PCR reactions were confirmed by sequencing and BLAST analysis [25] conducted by the National Center for Biotechnology Information (NCBI).

Virus isolation was performed according to the methodology in reference [22].

### Virus separation

Allantoic fluid derived from the 2^nd^ passage of the co-infected sample P/18K (hemagglutination test titer of 128) positive for both the H6 and H11 in RT-PCR was serially diluted in a range of 10^−1^ to 10^−4^ in distilled water. Then, 100 µl of each dilution series were mixed with 100 µl of H6 or H11 antibodies and incubated for 30 min at room temperature. Subsequently, 200 µl of allantoic fluid-antibody mixture for each dilution was inoculated into the allantoic sac of two specific pathogen free (SPF) embryonated chicken eggs and incubated at 37°C. After embryo death, the allantoic fluid was recovered and tested by the hemagglutination test [22]. Finally, the H6, H11, N2, and N9 subtypes were determined via RT-PCR assays according to the above described procedures.

### Sequencing analysis

Partial or whole genome amplification (OneStep RT–PCR kit, Qiagen) was performed with various combinations of the previously described primers [21], [24], [26]–[28] and primers from our primer library selected from the conserved and semiconserved regions of each genomic segments of the IAV (available on request). If needed, the second PCR round was carried out using the same primer combination. The amplification products of expected size were purified or cut from the agarose gel and purified by the High Pure PCR Product Purification Kit (Roche) and sequenced using the BigDye Terminator Cycle–Sequencing Ready Reaction Kit version 3.1 (Life Technologies). Besides the primers used for the amplification, sequencing primer sets (available on request) have also been employed to ensure full-amplicon or full-coding sequence read and increase the position coverage. Sequence analysis was performed on a 3130 genetic analyzer (Life Technologies). The particular sequence positions were covered 3-times on average.

### Phylogenetic analysis and genotyping

The sequences were assembled and edited and the sequence quality was evaluated by the SeqScape software (Life Technologies). BLAST analysis [25] was then performed for all segments of each individual isolate across the NCBI database. The sequences were aligned with the MAFFT program (Multiple Alignment using Fast Fourier Transformation) [29]. Subsequently, alignment trimming, and sequence identity matrix and sequence difference count matrix calculation at the nucleic and amino acid levels were performed using the BioEdit 7.0.9.0 program [30]. Maximum likelihood (ML) trees were calculated using the MEGA software version 6.0 [31]. For each genomic segment the best nucleotide substitution model was inferred on the basis of the lowest Bayesian Information Criterion and Akaike information criterion scores. According to these selection procedures the following models were implemented: Hasegawa-Kishino-Yano + discrete Gamma distribution with 5 rate categories (HKY+G for PB2, PB1, PA, H6, and H11 sequences), Kimura 2-parameter +5G (K2+G for NP and MP sequences), and Tamura 3-parameter (T92 for N2, N9, and NS sequences). The robustness of nodes was evaluated by performing 1000 bootstrap replicates. Trees were drawn by the TreeExplorer tool in the MEGA 6.0 program. For phylogenetic analysis of the H11, N2, and N9 segments, which were represented only by a few amplicons, the data was supplemented with sequences from BLAST hits as well as additional IAV sequences of interest.

The results of phylogenetic analyses were summarized by employing the digital genotyping approach [32].

### GenBank submission

The sequences were deposited in GenBank with the accession numbers listed in Table 1.

**Table 1:** avian IAV strains characterized in our study.

Virus Name	Subtype	Cq	Virus isolation	Abbreviation	Genome sequencing status	GenBank Acc. no.	Number
A/mallard/CZE/15902-3K/09	N9	33.8	No	P/3K	partial: NP, NS	KC599271-73	1
A/mallard/CZE/15902-4K/09	H6	34.3	No	P/4K	partial: PB1, H6, MP, NS	KC599274-76	2
A/mallard/CZE/15902-9K/09	nd	35.5	No	P/9K	partial: PB2, PB1, PA, MP, NS	KC599277-80	3
A/mallard/CZE/15902-12K/09	H11N2	36.8	No	P/12K	partial: PB1, PA, H11, NP, N2, MP, NS	KC599281-87	4
A/mallard/CZE/15902-14K/09	H6N2	29.7	No	P/14K	partial: PB2, PB1, PA, H6, NP, MP, NS	KC599288-94	5
A/mallard/CZE/15902-17K/09	H6N2	21.3	Yes	P/17K	entire coding genome	HQ244427-34	6
A/mallard/CZE/15902-18K/09++	H11N9	25.8	Yes	P/18K_H11	entire coding genome	JF682618-25	7
	H6N9			P/18K_H6	entire coding genome	KC599295-302	8
A/mallard/CZE/15902-23K/09	H6N2	29.6	No	P/23K	each segment partial	KC599309-16	9
A/mallard/CZE/15902-25K/09	H11N9	31.0	No	P/25K	each segment partial	KC599317-24	10
A/mallard/CZE/15902-18T/09++	H6	32.4	No	P/18T	partial PB2, PB1, PA, H6, NP, MP, NS	KC599303-08	11
A/mallard/CZE/15962-1T/10	H6N9	27.4	No	H/1T	entire coding genome	JQ737234-41	12
A/mallard/CZE/15962-4T/10	H6N9	26.6	No	H/4T	each segment partial	KC599325-32	13

The number assigned for each strain corresponds to Figure 2.

CZE, Czech Republic.

T, tracheal swab.

K-cloacal swab.

P, H sampling localities (see the Figures S1 and S2 in File S1).

nd, not determined.

++ samples 18K and 18T did not originate from the same organism.

The Cq values correspond to the reference 20.

## Results

### Background information

Cloacal and tracheal swabs from mallards (Anas platyrhynchos) inhabiting a pond in the South Bohemian Region (further referred to as locality P, Figure S1 in File S1) and harvested by hunters on 30 September 2009 were investigated for avian IAV during the National avian IAV surveillance program in the Czech Republic. Of 25 cloacal (K) and 25 tracheal (T) swabs, 10 (nine cloacal and one tracheal) were RT-qPCR positive. Subsequent analysis revealed the presence of three different IAV subtypes: H6N2 (14K, 17K, 23K), H11N9 (25K), and H11N2 (12K). One sample (18K) showed H6, H11, and N9 positivity. Three swabs (3K, 4K, and 18T) were partially subtyped as N9, H6, and H6, respectively, and for one specimen (9K) the HA and NA subtypes were not determined. No H6N9 subtype was detected, even by means of repeated RT-PCR. Virus isolation was successful from two cloacal swabs (nos. 17 and 18). From swab no. 17K the H6N2 virus was retrieved (P/17K) whereas the allantoic fluid derived from swab no. 18K exhibited successful co-isolation of both H6 and H11 subtypes (P/18K).

One year later, on 1^st^ November 2010, avian IAV of H6N9 subtype was detected in mallards residing in a pond (further referred to as locality H, (Figure S2 in File S1)) located approximately at 25 km from the previous sampling area. Of 20 cloacal and 20 tracheal swabs, 6 were RT-qPCR positive. Two tracheal swabs (nos.1T and 4T) exhibited H6N9 subtype positivity and the remaining four were not subtyped. Virus isolation was not successful. Preliminary analysis of the H6 and N9 sequences revealed unusually high sequence identity with IAV strains from the locality P.

The ponds P and H belong to the artificially established and densely distributed pond systems of South Bohemia and are not interconnected with streams. These two ponds were managed by different hunting associations rearing mallards for hunting purposes. In both areas, the mallard population undergoes an annual de- and re-population cycle as follows: during spring, 3 week-old ducklings are bought and raised indoors for two-four weeks. At the age of five or six weeks, the birds receive a vaccine shot against *Clostridium botulinum* and are released to the respective ponds. They are kept outdoors until the hunting harvesting takes place in autumn. Next year the entire re- and de-population cycle repeats. The hunting organizations were not in mutual contact and the ducklings for the two ponds were bought independently from different suppliers. The bank of pond P was lined with wooden huts attracting wild mallards to nest (Figure S1b in File S1). The mallard population on pond P in 2009 was 500 birds and that on pond H in 2010 was 400 birds.

### Sequence and phylogenetic analysis

**Figure 1:**
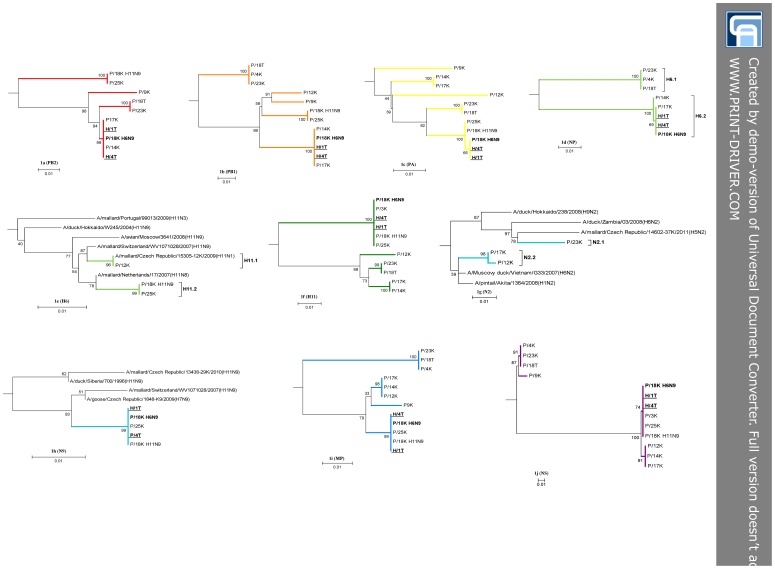
Phylogenetic trees of the locality P and H avian influenza viruses. Trees were generated with maximum-likelihood method in the MEGA 6.0 software on the basis of nucleotides 1251–2288 (1038) of PB2, 1465–2289 (825) of PB1, 783–1401 (649) of PA, 816–1724 (909) of H6, 679–1274 (596) of H11, 748–1544 (797) of NP, 568–889 (322) of N2, 1050–1431 (382) of N9, 203–1006 (804) of MP, and 540–870 (331) of NS. The nucleotide substitution models implemented were listed in the Materials and methods section. Bootstrap values (1000 re-samplings) in percentages are indicated at each node. The locality P H6N9 strain (P/18K_H6N9) was highlighted in bold and the locality H H6N9 strains (H/1T and H4/T) are bold and underlined. Each particular tree was supplemented with a nucleotide sequence identity matrix table (Tables S3a–j in File S1). The sub-clades of interest were highlighted with a segment specific color which is corresponding to Figure 2 and the abbreviations used with Table 1 respectively.

The presented unique spatial, temporal, and IAV subtype setting prompted us to investigate the sequence and genomic relationships within and between the locality P and H viruses in more detail. To this end, partial or entire coding genome sequencing was performed and the phylogenetic relationships and nucleotide and amino acid sequence differences between the respective genome segments were inferred. Since our IAV pool contained two different HA and NA subtypes, phylogenetic trees were constructed for all of them (Figures 1a–j). The sequencing status of each specimen included in the analysis is summarized in Table 1.

Overall, the phylogenetic analysis of the locality P viruses revealed remarkable sequence diversity. Depending on the genome segment, up to five distinct sub-lineages with significant bootstrap support were identified. The number of sub-lineages decreased from five (PB1 and PA) to four (PB2, NP, and MP) and three (NS); (Figures 1a–c, f, i, j). In addition, the NS segment was represented by both of the two alleles. Interestingly, remarkable sequence diversity was also observed within the H6, N2, and H11 trees with two recognizable sub-clusters designated as 1 and 2 (Figures 1d, e, and g). This indicated deeper complexity and apparent co-circulation of two distinct H6 and H11 genotypes within the same sampling locality. The N9 tree did not show discrete clustering of the sequences investigated (Figure 1h).

The nucleic acid sequence alignment of the regions used for phylogeny estimation showed diversity with peaks in a range from 5.1 to 7.5% between the segments, namely: PB2 (78/1038; 7.5%), PB1 (60/825; 7.3%), PA (33/649; 5.1%), NP (55/797; 6.9%), MP (41/804; 5.1%). Regarding the segments encoding for the surface antigens, the major differences were identified within the H6 (61/909; 6.8%), N2 (21/322; 6.6%) and H11 (12/596; 2%) sequences. The N9 amplicons were identical at the nucleotide sequence level (Tables S3a–j in File S1).

The high nucleotide variation contrasted with the high identity at the amino acid sequence level. The differences spanned below 6 residues within the investigated regions regardless of the segment considered. The only exception was the NS segment with 18 amino acid differences in the NS1 and 21 between the NS2 protein fragments, respectively, which corresponds to the known dual allelic structure in avian viruses.

Contrary to the high genetic diversity within locality P, the phylogenetic analysis of the H6N9 strains (locality H) did not reveal any discrete sequence clustering and the two representative genomes were 100% identical at the nucleotide sequence level (Tables S3a–d, f, h–j in File S1).

Finally, we established the relationships between the IAV strains from localities P and H both in terms of phylogenetic analysis and sequence identity. The results of phylogenetic analyses revealed that the PB2, PB1, and H6 segments of the H/H6N9 strains were closely related to those of the P/H6N2 sub-lineage 2 viruses while the PA, NP, N9, MP, and NS segments clustered within the P/H11N9 sub-lineage 2. This was further supported by almost 100% nucleotide sequence identity between the H/H6N9 and corresponding P/H6N2 and P/H11N9 segments (Tables S3a–d, f, h–j in File S1).

### Digital genotyping

**Figure 2:**
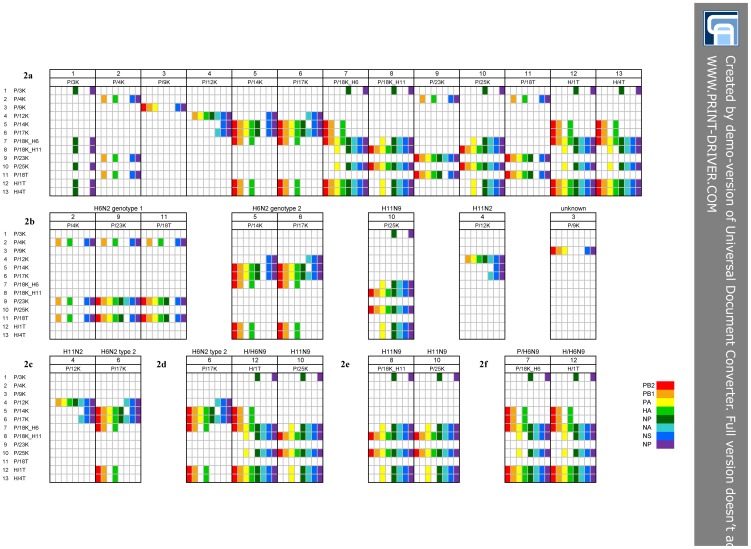
Segment identity matrix (SIM). The SIM was generated by plotting the influenza A virus (IAV) genomes against each other, with the relationships between the segments derived from the phylogenetic trees (Figure 1a–j) highlighted with colored pixels. The virus nomenclature corresponds to that in Table 1. The deduced genome constellations in the SIM were represented by columns 1–13 and the pixels within the columns were aligned according to the conventional listing of the IAV genome segments (from left to right: PB2, PB1, PA, HA, NP, NA, MP, and NS). The color scheme for the segments is given at the bottom of the figure and corresponds to the tables S3 in File S1. Empty pixels mean unknown or undetermined. Figures: 2a, the entire SIM; 2b overview of the genomic diversity of locality P IAV. For information regarding the Figures 2c-f please refer to the text.

The summarization of the results of phylogenetic analysis within the segment identity matrix (SIM) revealed at least five distinct IAV genotypes which co-circulated in locality P (Figure 2b): H6N2 genotype 1 (columns 2, 9, and 11), H6N2 genotype 2 (columns 5 and 6), H11N9 (column 10), H11N2 (column 4), and one with unknown subtype (column 3). Except the H11N2 and H6N2 genotype 2 viruses, which shared identical N2, MP and NS sequences (Figure 2c), no additional reassortment was observed. On the other hand, the two H6N9 strains 1T and 4T, representing locality H, had identical genome constellations (Figure 2a, columns 12 and 13).

Finally, the mutual comparison of the SIM columns derived from locality P and H IAV genomes clearly showed that the H/H6N9 genome was assembled from the P/H6N2 genotype 2 and P/H11N9 viruses at a 3∶5 ratio (Figure 2d). Indeed, the PB2, PB1, and H6 segments of the H/H6N9 genome were acquired from the P/H6N2 genotype 2 viruses while the PA, NP, N9, MP, and NS segments from the P/H11N9 strain.

The results of phylogenetic analysis and digital genotyping indicated that the H/H6N9 strains detected in 2010 were apparently direct progenies of the previously co-circulating P/H6N2 genotype 2 and P/H11N9 viruses.

### Investigation of co-infection

The close genetic relationships uncovered between H6N9 viruses from localities P and H drew our attention back to the H6/H11 co-infected cloacal swab specimen P/18K and to the IAV strains retrieved by virus isolation on ECE from that specimen.

First of all, we focused on the allantoic fluid and estimated the IAV subtype composition by the H6, H11, N2, and N9 specific RT-PCR tests. Surprisingly, the results revealed positivity for all segments but N2, which indicated that the allantoic fluid of P/18K evidently represented a mixed population of the H6N9 and H11N9 subtypes. Subsequently, these subtypes were successfully separated using H6 and H11 subtype-specific monoclonal antibodies and confirmed by RT-PCR tests and sequencing.

In the next step, we inferred the genomic constellation of the antibody separated H11N9 and H6N9 IA viruses. Summarization of the sequencing results of the entire coding genome clearly demonstrated that the entire genome of the P/18K_H11N9 virus was identical to that of another P/H11N9 strain, P/25K (Figure 2e). This H11N9 strain was also identified as a putative five-segment donor of the H/H6N9 strains. Finally, genotyping of the antibody separated P/18K_H6N9 virus revealed identity between the P/18K_H6N9 and H/H6N9 strains, again supported by *de facto* 100% similarity at the nucleotide sequence level (Fig 2f).

Taking together all these data, since only one parental virus (P/H6N2 genotype 2, Table 1) was successfully isolated, the antibody separation approach led to the retrieval of the second parental H11N9 strain as well as the progeny H6N9 strains.

Finally, having the H6N9/H11N9 mixed allantoic fluid analyzed, the subtype composition of the original cloacal swab specimen was inspected employing the N2 specific RT-PCR test. The result was essentially the same like in the allantoic fluid case, i.e. the absence of the N2 segment. This indicated that the parental subtype H6N2 was apparently not present in this cloacal swab either. Thus, the original status of the co-infected cloacal specimen P/8K was H11N9 and H6N9 positive.

## Discussion

Direct relationships between IAV from localities P and H were first indicated during the analysis of the H6N9/2010 IAV from locality H. The sequences obtained were first compared to our data containing genome sequences from various avian IAV detected in the Czech Republic between 2007 and 2011. This preliminary analysis showed absolute sequence identity to the viruses detected one year before in nearby locality P.

Detailed analysis of the specimens collected from locality P revealed co-circulation of four subtype combinations, H6N2, H6N9, H11N2, and H11N9, and six genomic constellations four of which were entirely different. For two subtypes, H6N9 and H11N2, the reassortment pattern was indicated. Among the H6, H11, and N2 segments, two sub-clades could have been clearly recognized. One specimen showed co-infection with two sub-types, H11N9 and H6N9.

Co-circulation of four entirely different IAV genotypes could theoretically led to double, triple, or even quadruple co-infections with a potential to generate 2^8^ or even as many as 4^8^ genomic constellations. This suggests, considering the high compatibility of the genomic segments [8] and the immunological naivety of the mallards, an IAV genotype explosion in locality P. Nevertheless, such extreme genomic diversity was not observed. In addition, the co-infection prevalence was lower than estimated previously [33], [34]. From this point of view, it is reasonable to suppose that the IAV genomic diversity in locality P might have been greater than observed and that the disproportions presumably resulted from multiple factors like the small number of samples collected (representing only 5% of the birds reared in pond P) or the sampling bias relative to the culmination of the infection, as well as from additional environmental variables. Despite these limitations, our data clearly indicated that the IAV genetic diversity at a local-scale level can be unexpectedly complex which adds new evidence to the recent study of Wille and colleagues [11]. In addition we demonstrated that thorough sequence analysis and genotyping could reveal the most intimate genetic links and infer the very recent reassortment events between the co-circulating IAV strains. Therefore, obtaining deeper insight into the diversity and dynamics of IAV at the local-scale would require long-term monitoring efforts targeted on the same locality with using the advantage of sentinel birds [10], [12] preferably in combination with parallel wild bird sampling [11] and followed by a detailed genotype analysis of all detected IAV strains.

The analysis of the co-infected specimen from locality P showed the presence of two HA subtypes, H6 and H11, and a single NA subtype, N9. This constellation was observed both in the screened allantoic fluid and the original swab material. We used specific H6 and H11 antibodies to separate these two subtypes. A similar strategy was applied previously to investigate IAV co-infections in wild ducks [33] although with a different experimental protocol. To this end, the allantoic fluid from the 2^nd^ passage was used as a starting material because the primary culture and the first passage were overgrown with bacterial contamination. This approach led to successful separation of the H11N9 and H6N9 viruses. Subsequent analyses revealed almost absolute sequence identity between the respective segments of the antibody separated and, let's say, native counterparts (P/H11N9, P/H6N2, and H/H6N9). This sufficiently proved that, contrary to the previous observations of Lindsay and colleagues [35], in our specific case the two ECE passages did not alter the genomic status of the co-infecting viruses in terms of *in vitro* reassortment and ruled out artificial generation of the P/H6N9 subtype during the virus isolation efforts. So, the co-infected specimen evidently contained both the H6N9 and H11N9 viruses.

Two conclusions can be drawn regarding the emergence of the H6N9 virus: i) the H6N9 subtype was evidently present, if not originated, in locality P, ii) the H6N9 virus persisted in the same area as was suggested by its re-detection roughly one year apart in the nearby locality H.

Genotyping of the antibody separated P/H6N9 virus showed that it was a 3∶5 reassortant of the P/H6N2 genotype 2 and P/H11N9 viruses with almost 100% identity of the respective genome segments at the nucleotide sequence level. At first sight, it indicates that the P/H6N9 virus represents a possible progeny of the P/H6N2 genotype 2 and P/H11N9 viruses. However, considering the close co-circulation of these viruses, it was not possible to determine which one is the parent and which one is the progeny. Furthermore, it is not clear whether the P/H6N9 virus emerged within the co-infected mallards or originated from elsewhere and subsequently co-infected the same bird along with the related (parental) P/H11N9 strain. Nevertheless, the genotype constellations favor the suggested parent-progeny scenario.

Despite a roughly one year interval between the P/H6N9 and H/H6N9 detection, both of the viruses retained identical subtype and genotype constellation. Unexpectedly, the entire genomes exhibited almost absolute nucleotide sequence identity. Considering the rapid evolutionary dynamics of avian IAV [9], the one-year interval between the two H6N9 strains should mean at least 13 nucleotide differences. Such or higher discrepancies between the isolation dates and unexpectedly high genetic conservation were previously attributed to laboratory artifacts [18], [19]. Vertical audit of our entire virus isolation, amplification, sequencing, and sequence assembly procedure unequivocally excluded contamination or data misinterpretation. In addition, the P and H/H6N9 viruses were sequenced and analyzed one year apart and, in the meantime, various additional and unrelated avian IAV were isolated and sequenced by using the same primer sets and reagents.

So, which mechanism would account for the exceptionally high sequence conservation of the H6N9 virus? To address this question, we performed epizootological investigations which included visiting the sample collection sites as well as communication with the hunting association representatives and field veterinarians who assisted in specimen collection. The investigation excluded any mutual contacts, cooperation, trade or involvement of other man-associated routes to allow artificial transmission, one-year preservation, and re-appearance of the H6N9 virus. Therefore, it is reasonable to hypothesize that the observed conservation of the H6N9 virus resulted from its environmental persistence and frozen evolution. We suppose that some of the H6N9 infected birds in locality P might have been frightened by hunters and escaped to nearby pond H where they disseminated the virus into the environment. Then, the H6N9 strain persisted in the environment and infected the new and immunologically naive mallard flock re-populating pond H next-year.

The frozen evolution is long considered as one of the mechanisms of influenza virus perpetuation in nature [13]. Nevertheless, its significance in the IAV ecology is not fully understood. In addition, there is no consistent view on this phenomenon in the literature [13]–[16], [18], [19]. In a recent study Shoham and colleagues [36] have demonstrated the ability of productive year-to-year preservation of avian IAV in arctic and sub-arctic ice which indicates that the frozen evolution evidently might operate in nature. However, we observed this phenomenon in the temperate zone. Similarly, Globig and colleagues [10] reported avian H3N2 strains with unusually similar HA and NA sequences detected roughly three months apart in sentinel mallards kept at a pond in Southern Germany which is in a 500 km distance from our sampling localities. This finding also supports our observations that besides extensive genetic variation, the frozen evolution and re-appearance of identical or unusually similar IAV strains may apparently act as an additional mechanism of virus perpetuation in wild aquatic birds. Again, additional and more complex surveillance efforts are required to fully elucidate this phenomenon.

It has been thought that IAV do not prevail in the form of latent infection in birds. In addition, pond H underwent annual re and de-population cycles. Hence, the genetic conservation of the H6N9 virus suggests some kind of environmental persistence. This raised another important question: On which environmental matrix could the virus persisted? The H6N9 virus had to survive through winter, spring, and especially summer, which is relatively hot in the temperate zone, to re-appear during the autumn. Although recent data suggests that unfavorable environmental conditions during summer do not prevent circulation of avian IAV in the environment [37] apparently none of the abiotic reservoirs of avian IAV studied so far like feces, intact pond water, or pond sediments [38]–[41] provide protection for a sufficiently long time period to ensure year-to-year preservation of the virus in our climatic zone. Moreover, the environmental persistence and subsequent productive re-infection requires preservation in a sufficiently concentrated state to prevent progressive dilution. This further argues against the majority of the abiotic components. Similarly, the significance of the biotic environmental components as long term reservoirs is also negligible [42]–[47].

Nevertheless, a mechanism has been characterized to date which can fit our assumptions. It has been demonstrated that feathers covered with preen oil could efficiently capture and concentrate the avian IAV from water [48]. Subsequently, the virus particles adsorbed on bird's bodies may mediate infection through self-preening or allo-preening activities. Although feather swabs collected from experimentally preened birds were positive by virus isolation roughly for one month [49] it is not known how long the virus can survive in preened feathers. Can the hydrophobic preen oil on detached feathers provide a sufficiently protecting environment for between-year persistence?

Although our conclusions probably raise more questions than they answer the results of the presented study suggest that the IAV subtype and genotype diversity between the IAV at the local-scale level can be admirably complex. Evaluation of the most intimate genetic links between such viruses can reveal rarely observed phenomena like direct parent-progeny relationships between the co-circulating strains or frozen evolution. Further and more detailed studies are required to fully elucidate whether between year persistence and frozen evolution observed here is an isolated unique event or represents a more regular but yet unrecognized phenomenon in the evolution of the influenza virus in aquatic birds.

## Supporting Information

File S1 **Figure S1. Locality P.** The samples in 2009 were collected from mallards inhabiting the pond called Putim (GPS coordinates 49°16′42.629″N, 14°8′9.881″E). The green flag on the map S1c represents the position of the S1a view. Figure S1b shows wooden huts distributed along the shore. The map was generated by the www.mapy.cz. **Figure S2: Locality H.** The samples in 2010 were collected from mallards resided the pond called Kahoun which is situated near the Hajany village (GPS coordinates 49°26′59.547″N, 13°49′52.568″E). The green flag on the map S2b represents the position of the panoramic view S2a. The map was generated by the www.mapy.cz. **Table S3: Nucleic acid sequence identity matrices of the locality P and H avian influenza virus segments.** The matrices were constructed using the BioEdit program on the basis of nucleotides 1251–2288 (1038) of PB2, 1465–2289 (825) of PB1, 783–1401 (649) of PA, 816–1724 (909) of H6, 679–1274 (596) of H11, 748–1544 (797) of NP, 568–889 (322) of N2, 1050–1431 (382) of N9, 203–1006 (804) of MP, and 540–870 (331) of NS. The tables were highlighted with a segment specific color which is corresponding to Figures 1 and 2 and the abbreviations with Table 1 respectively.
(PDF)
