# Supplementary material for: Local-Scale Diversity and Between-Year “Frozen Evolution” of Avian Influenza A Viruses in Nature
Source: PLoS One. 2014 Jul 30;9(7):e103053. doi: 10.1371/journal.pone.0103053 (PMC4116140; doi:10.1371/journal.pone.0103053)
Supplement: File S1 — Figure S1. Locality P. The samples in 2009 were collected from mallards inhabiting the pond called Putim (GPS coordinates 49°16′42.629″N, 14°8′9.881″E). The green flag on the map S1c represents the position of the S1a view. Figure S1b shows wooden huts distributed along the shore. The map was generated by the www.mapy.cz. Figure S2: Locality H. The samples in 2010 were collected from mallards resided the pond called Kahoun which is situated near the Hajany village (GPS coordinates 49°26′59.547″N, 13°49′52.568″E). The green flag on the map S2b represents the position of the panoramic view S2a. The map was generated by the www.mapy.cz. Table S3: Nucleic acid sequence identity matrices of the locality P and H avian influenza virus segments. The matrices were constructed using the BioEdit program on the basis of nucleotides 1251–2288 (1038) of PB2, 1465–2289 (825) of PB1, 783–1401 (649) of PA, 816–1724 (909) of H6, 679–1274 (596) of H11, 748–1544 (797) of NP, 568–889 (322) of N2, 1050–1431 (382) of N9, 203–1006 (804) of MP, and 540–870 (331) of NS. The tables were highlighted with a segment specific color which is corresponding to Figures 1 and 2 and the abbreviations with Table 1 respectively. (PDF) [file pone.0103053.s001.pdf]

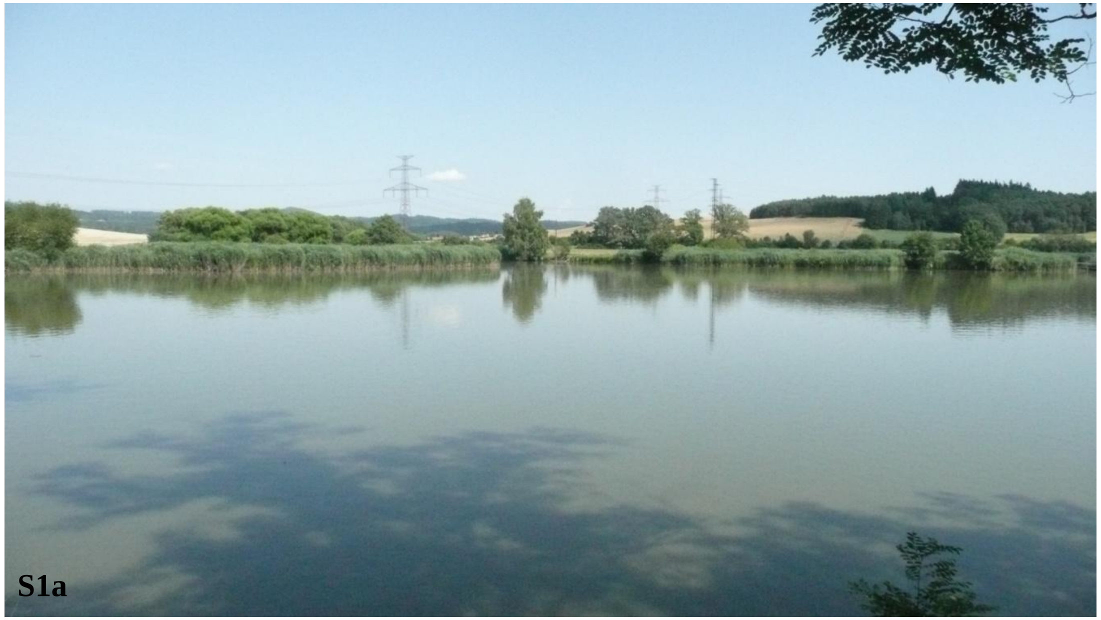

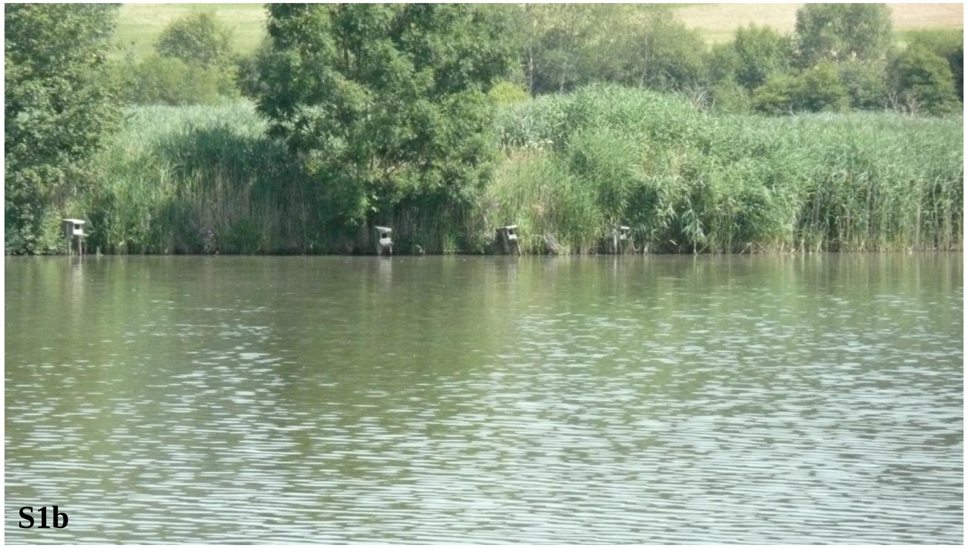

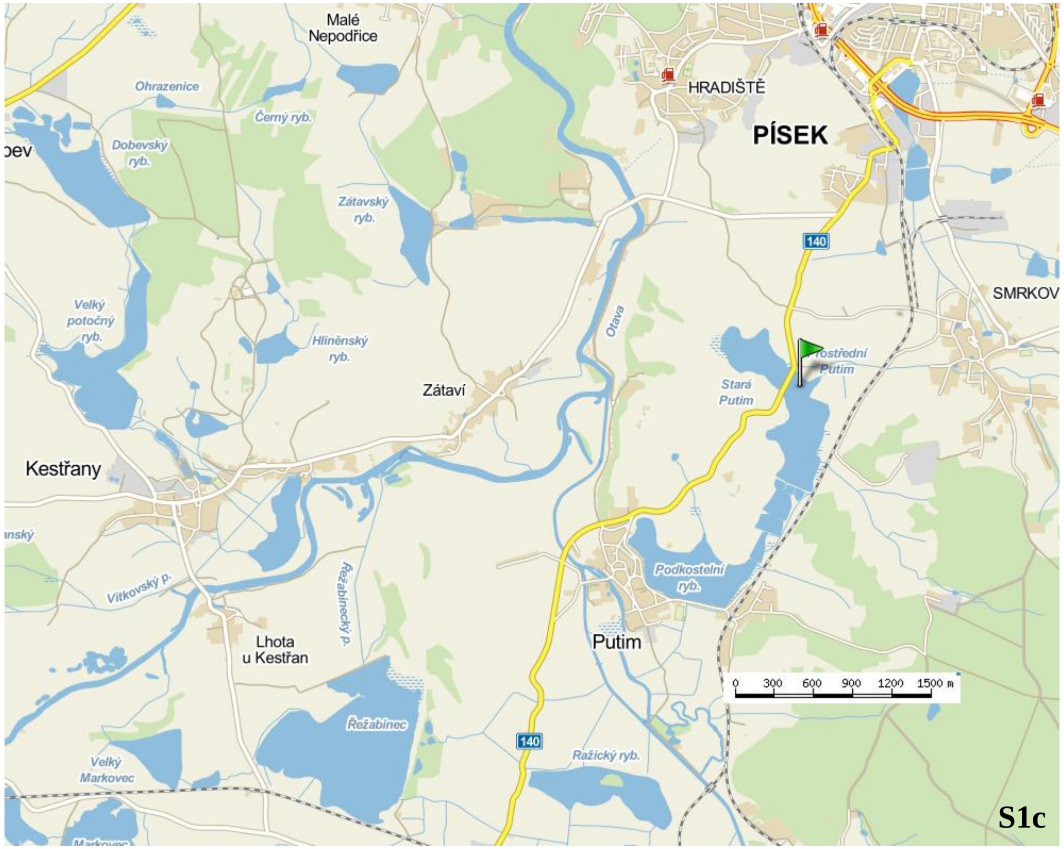

Figure S1: Locality P. The samples in 2009 were collected from mallards inhabiting the pond called Putim (GPS coordinates 49°16'42.629"N, 14°8'9.881"E). The green flag on the map S1c represents the position of the S1a view. Figure S1b shows wooden huts distributed along the shore. The map was generated by the www.mapy.cz.

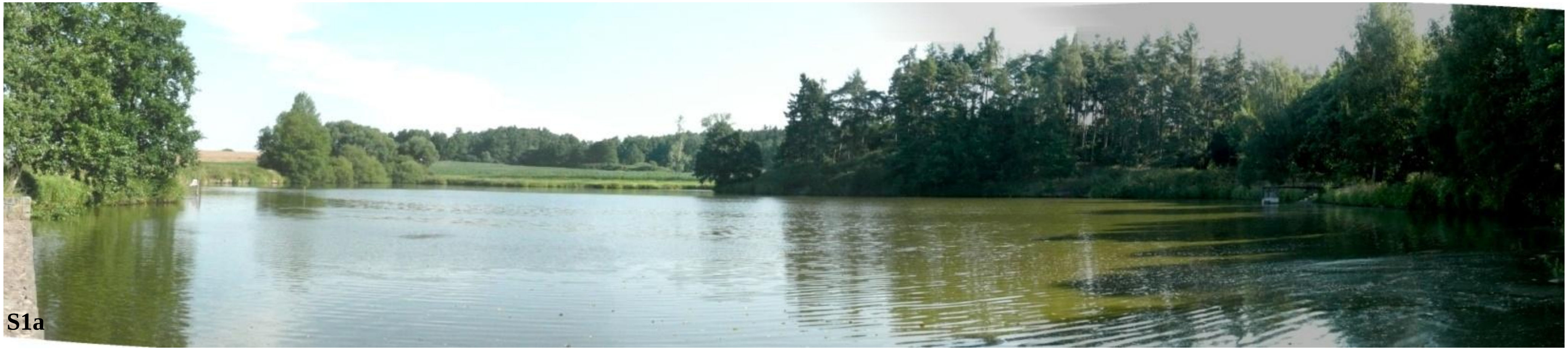

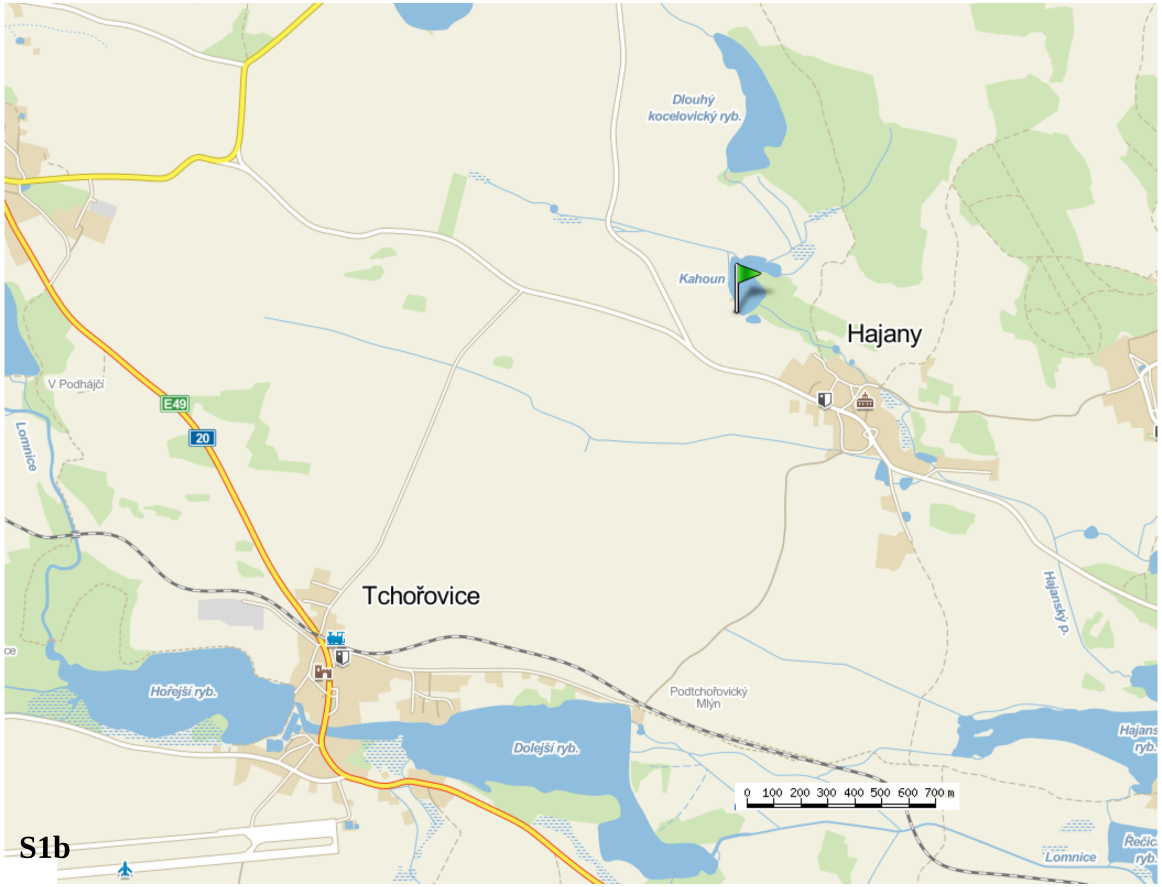

Figure S2: Locality H. The samples in 2010 were collected from mallards resided the pond called Kahoun which is situated near the Hajany village (GPS coordinates 49°26'59.547"N, 13°49'52.568"E). The green flag on the map S2b represents the position of the panoramic view S2a. The map was generated by the www.mapy.cz.

S3a: **PB2**

PB2 1038nt	P/9K	P/14K	P/17K	P/18K H6	P/18K H11	P/23K	P/25K	P/18T	H/1T	H/4T
P/9K	ID	28	28	28	78	40	78	40	28	28
P/14K	28	ID	0	0	66	16	66	16	0	0
P/17K	28	0	ID	0	66	16	66	16	0	0
P/18K_H6	28	0	0	ID	66	16	66	16	0	0
P/18K_H11	78	66	66	66	ID	74	0	74	66	66
P/23K	40	16	16	16	74	ID	74	0	16	16
P/25K	78	66	66	66	0	74	ID	74	66	66
P/18T	40	16	16	16	74	0	74	ID	16	16
H/1T	28	0	0	0	66	16	66	16	ID	0
H/4T	28	0	0	0	66	16	66	16	0	ID

S3b: **PB1**

PB1 825nt	P/4K	P/9K	P/12K	P/14K	P/17K	P/18K H6	P/18K H11	P/23K	P/25K	P/18T	H/1T	H/4T
P/4K	ID	56	57	58	58	58	60	0	60	0	58	58
P/9K	56	ID	26	40	40	40	36	56	36	56	40	40
P/12K	57	26	ID	40	40	40	36	57	36	57	40	40
P/14K	58	40	40	ID	0	0	42	58	42	58	0	0
P/17K	58	40	40	0	ID	0	42	58	42	58	0	0
P/18K_H6	58	40	40	0	0	ID	42	58	42	58	0	0
P/18K_H11	60	36	36	42	42	42	ID	60	0	60	42	42
P/23K	0	56	57	58	58	58	60	ID	60	0	58	58
P/25K	60	36	36	42	42	42	0	60	ID	60	42	42
P/18T	0	56	57	58	58	58	60	0	60	ID	58	58
H/1T	58	40	40	0	0	0	42	58	42	58	ID	0
H/4T	58	40	40	0	0	0	42	58	42	58	0	ID

S3c: **PA**

PA 649nt	P/9K	P/12K	P/14K	P/17K	P/18K H6	P/18K H11	P/23K	P/25K	P/18T	H/1T	H/4T
P/9K	ID	33	25	25	29	27	29	27	29	28	28
P/12K	33	ID	26	26	30	28	30	28	30	29	29
P/14K	25	26	ID	0	24	22	20	22	20	23	23
P/17K	25	26	0	ID	24	22	20	22	20	23	23
P/18K_H6	29	30	24	24	ID	2	16	2	16	1	1
P/18K_H11	27	28	22	22	2	ID	14	0	14	1	1
P/23K	29	30	20	20	16	14	ID	14	0	15	15
P/25K	27	28	22	22	2	0	14	ID	14	1	1
P/18T	29	30	20	20	16	14	0	14	ID	15	15
H/1T	28	29	23	23	1	1	15	1	15	ID	0
H/4T	28	29	23	23	1	1	15	1	15	0	ID

S3d: **H6**

H6 909nt	P/4K	P/14K	P/17K	P/18K H6	P/23K	P/18T	H/1T	H/4T
P/4K	ID	60	61	61	0	0	61	61
P/14K	60	ID	1	1	60	60	1	1
P/17K	61	1	ID	0	61	61	0	0
P/18K_H6	61	1	0	ID	61	61	0	0
P/23K	0	60	61	61	ID	0	61	61
P/18T	0	60	61	61	0	ID	61	61
H/1T	61	1	0	0	61	61	ID	0
H/4T	61	1	0	0	61	61	0	ID

S3e: **H11**

H11 596nt	P/12K	P/18K H11	P/25K
P/12K	ID	12	11
P/18K_H11	12	ID	1
P/25K	11	1	ID

S3f: **NP**

NP 797nt	P/3K	P/12K	P/14K	P/17K	P/18K H6	P/18K H11	P/23K	P/25K	P/18T	H/1T	H/4T
P/3K	ID	55	53	53	0	0	55	0	55	0	0
P/12K	55	ID	21	21	55	55	18	55	18	55	55
P/14K	53	21	ID	0	53	53	11	53	11	53	53
P/17K	53	21	0	ID	53	53	11	53	11	53	53
P/18K_H6	0	55	53	53	ID	0	55	0	55	0	0
P/18K_H11	0	55	53	53	0	ID	55	0	55	0	0
P/23K	55	18	11	11	55	55	ID	55	0	55	55
P/25K	0	55	53	53	0	0	55	ID	55	0	0
P/18T	55	18	11	11	55	55	0	55	ID	55	55
H/1T	0	55	53	53	0	0	55	0	55	ID	0
H/4T	0	55	53	53	0	0	55	0	55	0	ID

S3g: **N2**

N2 322nt	P/12K	P/17K	P/23K
P/12K	ID	1	21
P/17K	1	ID	20
P/23K	21	20	ID

S3h: **N9**

N9 382nt	P/18K H6	P/18K H11	P/25K	H/1T	H/4T
P/18K_H6	ID	0	0	0	0
P/18K_H11	0	ID	0	0	0
P/25K	0	0	ID	0	0
H/1T	0	0	0	ID	0
H/4T	0	0	0	0	ID

S3i: **MP**

MP 804nt	P/4K	P/9K	P/12K	P/14K	P/17K	P/18K H6	P/18K H11	P/23K	P/25K	P/18T	H/1T	H/4T
P/4K	ID	39	39	39	39	41	41	0	41	0	41	41
P/9K	39	ID	10	10	10	14	14	39	14	39	14	14
P/12K	39	10	ID	0	0	10	10	39	10	39	10	10
P/14K	39	10	0	ID	0	10	10	39	10	39	10	10
P/17K	39	10	0	0	ID	10	10	39	10	39	10	10
P/18K_H6	41	14	10	10	10	ID	0	41	0	41	0	0
P/18K_H11	41	14	10	10	10	0	ID	41	0	41	0	0
P/23K	0	39	39	39	39	41	41	ID	41	0	41	41
P/25K	41	14	10	10	10	0	0	41	ID	41	0	0
P/18T	0	39	39	39	39	41	41	0	41	ID	41	41
H/1T	41	14	10	10	10	0	0	41	0	41	ID	0
H/4T	41	14	10	10	10	0	0	41	0	41	0	ID

S3j: **NS**

NS 331nt	P/4K	P/4K	P/9K	P/12K	P/14K	P/17K	P/18K H6	P/18K H11	P/23K	P/25K	P/18T	H/1T	H/4T
P/3K	ID	57	58	3	3	3	0	0	57	0	56	0	0
P/4K	57	ID	6	58	58	58	57	57	0	57	1	57	57
P/9K	58	6	ID	59	59	59	58	58	6	58	5	58	58
P/12K	3	58	59	ID	0	0	3	3	58	3	57	3	3
P/14K	3	58	59	0	ID	0	3	3	58	3	57	3	3
P/17K	3	58	59	0	0	ID	3	3	58	3	57	3	3
P/18K_H6	0	57	58	3	3	3	ID	0	57	0	56	0	0
P/18K_H11	0	57	58	3	3	3	0	ID	57	0	56	0	0
P/23K	57	0	6	58	58	58	57	57	ID	57	1	57	57
P/25K	0	57	58	3	3	3	0	0	57	ID	56	0	0
P/18T	56	1	5	57	57	57	56	56	1	56	ID	56	56
H/1T	0	57	58	3	3	3	0	0	57	0	56	ID	0
H/4T	0	57	58	3	3	3	0	0	57	0	56	0	ID

Table S3: Nucleic acid sequence identity matrices of the locality P and H avian influenza virus segments. The matrices were constructed using the BioEdit program on the basis of nucleotides 1251-2288 (1038) of PB2, 1465-2289 (825) of PB1, 783-1401 (649) of PA, 816-1724 (909) of H6, 679-1274 (596) of H11, 748-1544 (797) of NP, 568-889 (322) of N2, 1050-1431 (382) of N9, 203-1006 (804) of MP, and 540-870 (331) of NS. The tables were highlighted with a segment specific color which is corresponding to Figures 1 and 2 and the abbreviations with Table 1 respectively.
